# Enhancing predictive performance for spectroscopic studies in wildlife science through a multi-model approach: A case study for species classification of live amphibians

**DOI:** 10.1371/journal.pcbi.1011876

**Published:** 2024-02-14

**Authors:** Li-Dunn Chen, Michael A. Caprio, Devin M. Chen, Andrew J. Kouba, Carrie K. Kouba

**Affiliations:** 1 Department of Biochemistry, Molecular Biology, Entomology, & Plant Pathology, Mississippi State University, Mississippi, United States of America; 2 Department of Wildlife, Fisheries, & Aquaculture, Mississippi State University, Mississippi, United States of America; University of Virginia, UNITED STATES

## Abstract

Near infrared spectroscopy coupled with predictive modeling is a growing field of study for addressing questions in wildlife science aimed at improving management strategies and conservation outcomes for managed and threatened fauna. To date, the majority of spectroscopic studies in wildlife and fisheries applied chemometrics and predictive modeling with a single-algorithm approach. By contrast, multi-model approaches are used routinely for analyzing spectroscopic datasets across many major industries (e.g., medicine, agriculture) to maximize predictive outcomes for real-world applications. In this study, we conducted a benchmark modeling exercise to compare the performance of several machine learning algorithms in a multi-class problem utilizing a multivariate spectroscopic dataset obtained from live animals. Spectra obtained from live individuals representing eleven amphibian species were classified according to taxonomic designation. Seven modeling techniques were applied to generate prediction models, which varied significantly (p < 0.05) with regard to mean classification accuracy (e.g., support vector machine: 95.8 ± 0.8% vs. K-nearest neighbors: 89.3 ± 1.0%). Through the use of a multi-algorithm approach, candidate algorithms can be identified and applied to more effectively model complex spectroscopic data collected for wildlife sciences. Other key considerations in the predictive modeling workflow that serve to optimize spectroscopic model performance (e.g., variable selection and cross-validation procedures) are also discussed.

## Author summary

We explored the use of a multi-model approach for analyzing a spectroscopic dataset relevant to wildlife sciences. To date, the vast majority of spectroscopy studies for wildlife sciences have assessed spectral datasets with a single algorithm approach, but comparing multiple classification models may yield higher predictive outcomes for the given question and study system. This study utilized near-infrared spectra collected from the dermal surface of live amphibians across eleven species. We first demonstrate that NIR spectra collected from live amphibians produces unique spectral signatures characteristic of each species. Next, we evaluated the classification accuracy of seven machine learning algorithms for NIR spectra of unknown origin. Significant differences in performance accuracy were observed across model algorithms. With NIR data already digitized, a multi-model analysis may enhance predictive performance of existing or newly generated predictive models, which could lead to more robust as well as more informed decision-making regarding wildlife management practices based on spectroscopic data.

## Introduction

Near infrared (NIR) spectroscopy used in tandem with chemometric analysis is a non-destructive biophotonic technique that has recently gained traction for addressing questions in wildlife sciences [1]. Physiological traits can be discerned once models built from spectral data collected from biological samples (e.g., scent marks, feces, urine, hair, blood) or even the live animal, have been validated with direct methodologies [2]. NIR spectroscopy offers several advantages over standard single-trait assays by providing a high throughput of rich biochemical information, in the form of a biochemical signature captured in a digitized spectral profile. With technological advances, NIR spectrometers have become more portable and field-friendly providing on-the-ground, real-time predictive capability for assessing aspects of animal physiology, particularly in studies pertaining to wildlife demography (e.g., sex, age, count data), disease detection, reproductive status assessments, nutrition profiling, and species taxonomic determination [1]. The digitization of biochemical information captured in NIR spectra not only enables predictive model development across many applications at once, but also facilitates longitudinal studies that examine changes in population demographics or animal physiology and health over time [3]. Once prediction models have been generated, new data can be incorporated to enhance sampling variability and novel modeling approaches may also be applied to improve predictive modeling outcomes.

In the field of spectroscopy, chemometric analysis involving a wide array of mathematical and statistical operations can be employed to generate a prediction model for the biophysiological parameter of interest. There are numerous algorithms (also referred to as models or learners) each with their own associated tuning parameters that may be applied and tweaked to calibrate a prediction model [4]. In conventional classification and regression problems where the goal is to either predict the class or to quantify the value of an unknown sample for some target variable, unsupervised (e.g., k-means clustering, principal component analysis) and supervised learning (e.g., partial least squares, random forest, gradient boosting, Naïve Bayes) algorithms have been applied with great success [4]. Although commonly used chemometric software platforms (e.g., Unscrambler X, OPUS) are user-friendly and highly customizable in some respects they can be limited in the number of algorithms used for developing and testing models. At present, modeling algorithms such as linear discriminant analysis (LDA) and various versions of partial least squares (PLS) represent the two most commonly cited algorithms for generating spectroscopic prediction models in wildlife science (Table 1).

**Table 1:** Machine learning (ML) algorithms used in spectroscopic studies for wildlife science applications.

Vertebrate Class	Study Taxon (common name)	Target Question	Tissue Type Sampled	ML Algorithm*
**Fish**	Barramundi	Age	Otolith	PLS [3] PLS, MLR [22]
	Bass	Age	Vertebra	PLS [23]
	Cod	Age Spawning status	Otolith Ovarian tissue	PLS [3] PLS [24]
	Pollock	Age Spawning status	Otolith Ovarian tissue	PLS [3,25] PLS [24]
	Japanese rice fish	Embryo quality and development	Embryo body, oil droplets, yolk	LDA [26]
	Rockfish	Spawning status	Ovarian tissue	PLS [24]
	Salmon	Age	Otolith	PLS [3]
	Snapper	Age Embryonic development	Otolith	PLS [27, 28] PLS [3]
	Sharks	Age	Vertebra, dorsal fin spine, fin clip	PLS [23,29,30]
	Sturgeon	Follicular atresia	Live fish, ovarian follicles	SIMCA [31]
	Longnose skate	Age	Vertebra	PLS [3,32]
	Turbot	Spawning status	Ovarian tissue	PLS [24]
	Multiple species of fish	Species	Otolith	PLS [3] SIMCA, SVM, PLS, KNN [21]
**Amphibians**	Anurans	Sex Species	Skin of live individual Skin	PLS [33] LDA [34,35]
	Houston toad	Sex	Skin of live individual	LDA [19]
	Gopher frog	Sex Age	Skin of live individual	LDA [36] PLS [3]
	Chinese giant salamander	Sex	Skin of live individual	PLS [37]
	Dusky salamander	Pheromone expression	Skin of live individual	LDA [3]
**Reptiles**	Sea turtle	Nutrition	Stomach contents	PLS [38]
**Birds**	Macaw	Nutrition	Stomach contents	PLS [39]
	Ostrich	Nutrition	Excreta	PLS [40]
	Penguin	Myoglobin saturation Population ecology	Pectoral muscle implant Excreta	LMM [41] PLSR [42]
**Mammals**	Brown bear	Nutrition	Excreta	PLS [20]
	Giant panda	Nutrition Sex Pregnancy status	Plant leaves Excreta Excreta	PLS [43] PLS [44] PLS [45]
	Multiple species of ungulates	Nutrition	Hair	PLS [46]
	Pyrenean chamois	Nutrition	Excreta	PLS [47,48]
	Deer	Species Sex Stress	Excreta	PLS [49] PLS [50]
	Elk	Nutrition	Excreta Plant leaves	PLS, SMR [51] PLS [52,53]
	Gazelle	Nutrition	Stomach contents	PLS [54]
	Goat	Nutrition	Excreta	MPLS [55]
	Horse	Parasites	Excreta	PLS [56]
	Moose	Nutrition	Stomach contents	OPLS [57]
	Oryx	Nutrition	Excreta	PLS [58]
	African and Asian elephant	Nutrition Wildlife trade Species	Excreta Hair, Ivory Blood	PLS [59] PLS [60, 61] PLS [62]
	Giraffe	Wildlife trade	Hair	LDA [61]
	Rodent	Nutrition Species	Stomach contents, excreta Excreta	PLS [63] FDA [64]
	Amur leopard	Sex and species	Excreta	PLS, MDR [65]
	Snow leopard	Sex and species	Excreta	PLS, MDR [65]
	Indian leopard	Species	Blood traces	PLS [62]
	Bengal tiger	Species	Blood traces	PLS [62]
	Gorilla	Nutrition and food choice	Plant leaves	MPLS [66]
	Howler monkey	Sex and species	Hair	PLS [67]
	Orangutan	Estrus	Excreta	PLS [68]
	Dugong	Nutrition	Stomach contents, excreta, plant leaves	PLS [38,69,70]
	Pinniped	Nutrition	Excreta	PLS [71]
	Koala	Nutritional ecology	Plant leaves, excreta	PLS [72,73]
	Kangaroo	Nutrition Feeding ecology	Excreta Plant leaves, excreta	PLS [74] PLS [73]
	Hairy-nosed wombat	Nutrition	Plant leaves	PLS [75]
Multi-Class	Multiple species of fish, reptile, bird, mammal	Taxonomy	Skull Bones	PLS [76]

*Abbreviations for the algorithms applied for calibrating and testing models are as follows: KNN = K-nearest neighbors, LDA = linear discriminant analysis, LMM = linear mixing model, MDR = Mahalanobis distance discrimination based on PCA residuals, MLR = multiple linear regression, OPLS = orthogonal projection to latent structures, PLS = partial least squares, SIMCA = soft independent modeling of class analogies, SMR = stepwise multiple regression, and SVM = support vector machine.

However, there are many more algorithms for developing prediction models that are not only publicly available but are free to access in open-source software programs (e.g., Python, R). Given that an algorithm applies unique mathematical steps to generate predictions for the classification or regression task, it is entirely possible that different algorithms may offer more analytical flexibility, perform with varying levels of success, and some may prove to be better suited for the problem at hand [5–9]. Furthermore, the ability of an algorithm to perform a specific task may vary depending on the characteristics inherent to the dataset (e.g., number of observations vs. predictor variables) and study system (e.g., species, tissue type) being examined [4]. Overall predictive performance of spectroscopic-based models might benefit from a multi-model benchmark approach in which multiple algorithms are applied to independently train and test each of the models using identical data splits. Each model’s predictive performance is then compared to identify the candidate algorithm that best predicts the classification or regression task of interest. For example, model efficacy varied considerably when three supervised model procedures were utilized to detect papillary carcinoma from Raman spectra, such that the random forest (81.5%) and AdaBoost (84.6%) algorithms yielded markedly higher classification rates compared to the decision tree classifier (75.4%) [8]. While such multi-model approaches are routinely conducted to benchmark and evaluate candidate models in fields such as engineering, medicine, and agriculture [4,5,7,8,10–18], the majority of spectroscopic-based studies in wildlife sciences (61/65 studies; see Methods) have applied a single-algorithm approach for model calibration and testing (Table 1). PLS models have been applied to answer a diverse set of questions across many different sampling types, such as species discrimination from dried blood traces between Bengal tigers and Indian leopards, and quantification of dietary components from the feces of black bears [19, 20]. However, it may be that different sample types and associated questions can be better addressed through the exploration of other algorithms [21]. The wide variety of sampling modes (e.g., feces, hair, otolith, live animals) and questions addressed in wildlife sciences warrants a multi-faceted approach to robustly generate prediction models that are best suited for the sample type under investigation. For instance, K-nearest neighbors provided higher predictive outcomes compared to PLS when classifying structural differences among fish otoliths [21]. The current study aimed to test the utility of a multi-model benchmark procedure in a question relevant to wildlife sciences, specifically species classification using the spectra collected from the live tissue of amphibians. Species determination is a valuable demographic trait that can be used to inform management decisions based off evaluations of biodiversity and community dynamics.

To compare model algorithms, we first created a spectral database containing NIR spectra from several amphibian species belonging to two different orders, Anura (i.e., frogs and toads) and Caudata (salamanders and newts). Although the high water content present in the dermis and dermal secretions of amphibians is thought to weaken the quality of spectral signals, previous NIR spectroscopy work with amphibians found that biologically relevant information could be obtained from the surface of amphibian skin [33,35,37,77]. The porous, endocrine-like nature of amphibian skin allows the collection of a unique spectral profile that is likely associated with the skin’s biochemical composition and can be analyzed without the use of molecular-based (e.g., DNA analysis) or potentially invasive procedures [78]. The ability to hold, restrain, and scan amphibians in less than the minute needed to obtain spectra, makes them a valuable vertebrate class for studying how NIR spectroscopy can be used to obtain information on whole, live animals. Recent NIR studies on live amphibians have demonstrated promising results for evaluating and categorizing physiological factors such as biological sex, species, and reproductive status [19,36,37,77]. For example, taxonomic classification that previously required genetic sequencing or specialized field biologists to distinguish closely related anurans has been facilitated with NIR spectroscopy [35]. In the current study, we examined spectral profiles of eleven amphibian species to determine to what extent spectral profiles would differ between spectra belonging to different orders, families, genera, and species. We posited that species belonging to the same taxonomic clades (e.g., Order Anura or Caudata) would present greater similarities in their spectral profiles than species belonging to different taxonomic clades.

Secondly, we examined if predictive classification models based on the NIR spectra of amphibian skin in live amphibians could be improved through the employment of a multi-model benchmark approach. A benchmark modeling exercise was conducted in the open-source software, R, in a multi-class problem to compare the performance of seven conventional machine learning algorithms: generalized linear model with elastic net regression (GLM), K-nearest neighbors (KNN), linear discriminant analysis (LDA), partial least squares (PLS), random forest (RF), support vector machine (SVM), and extreme gradient boosting (XGB). The scope of this study is limited to the aforementioned algorithms, but many others have recently been applied (e.g., Naïve Bayes, neural networks) to generate prediction models for spectroscopic datasets [79–81]. Each model technique adheres to a unique, established set of mathematical rules to first assess spectral variability present in the dataset and then calibrate a model to subsequently make predictions on unseen test data. We investigated whether the seven algorithms applied would differ significantly with regard to their aggregated performance accuracies, as each model deploys a unique approach with varying characteristics (e.g., algorithm design, computational complexity, parameter tuning) to achieve the classification task of interest.

## Results

### The spectra, principal component loadings, & scores

**Fig 1:**
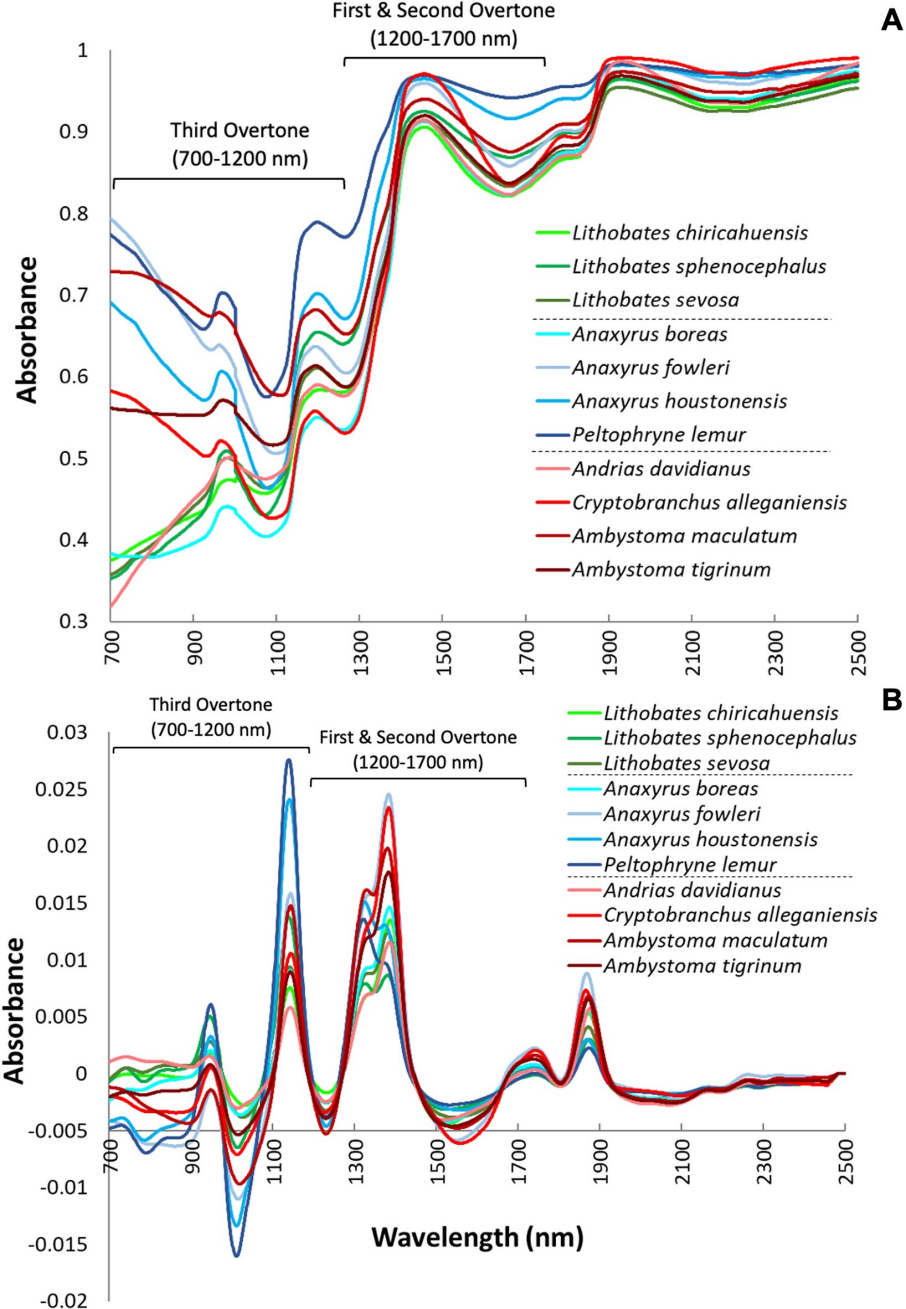
**A) Raw and B) transformed NIR absorbance spectra (700–2500 nm) were averaged and categorized according to their species designation**. The averaged spectra for each species are colored by taxonomic groupings: frogs = green, toads = blue, and salamanders = red. Distinct patterns can be observed between the eleven species investigated across several regions of the NIR spectrum.

Spectral data were averaged and categorized according to their species designation, which indicated visually distinct patterns between the eleven amphibian species investigated (Fig 1). More specifically, major differences between species can be observed in both the raw and transformed spectra on the third overtone region of the NIR spectrum spanning 700–1200 nm, as well as the first and second overtone regions of the NIR spectrum that correspond to the range between 1200–1700 nm (Fig 1A & 1B). Overall, marked variability in spectral patterns were observed between the amphibians examined, especially in the NIR region adjacent to the visible region ranging from 700–1000 nm. Several additional spectral features were revealed in the transformed spectra (Fig 1B). Within the first overtone region, there is a distinct water signal spanning 1300–1600 nm, where spectral patterns can be distinguished between taxonomic orders (Anura in green and blue vs. Caudata in red). Differing moisture signals among species are notable and should be considered when assessing the spectra obtained from live tissue. Additionally, the application of mathematical pretreatments (see Methods) resolved the noisiness of some peaks (e.g., at approximately 950 nm) and improved overall spectral resolution.

**Fig 2:**
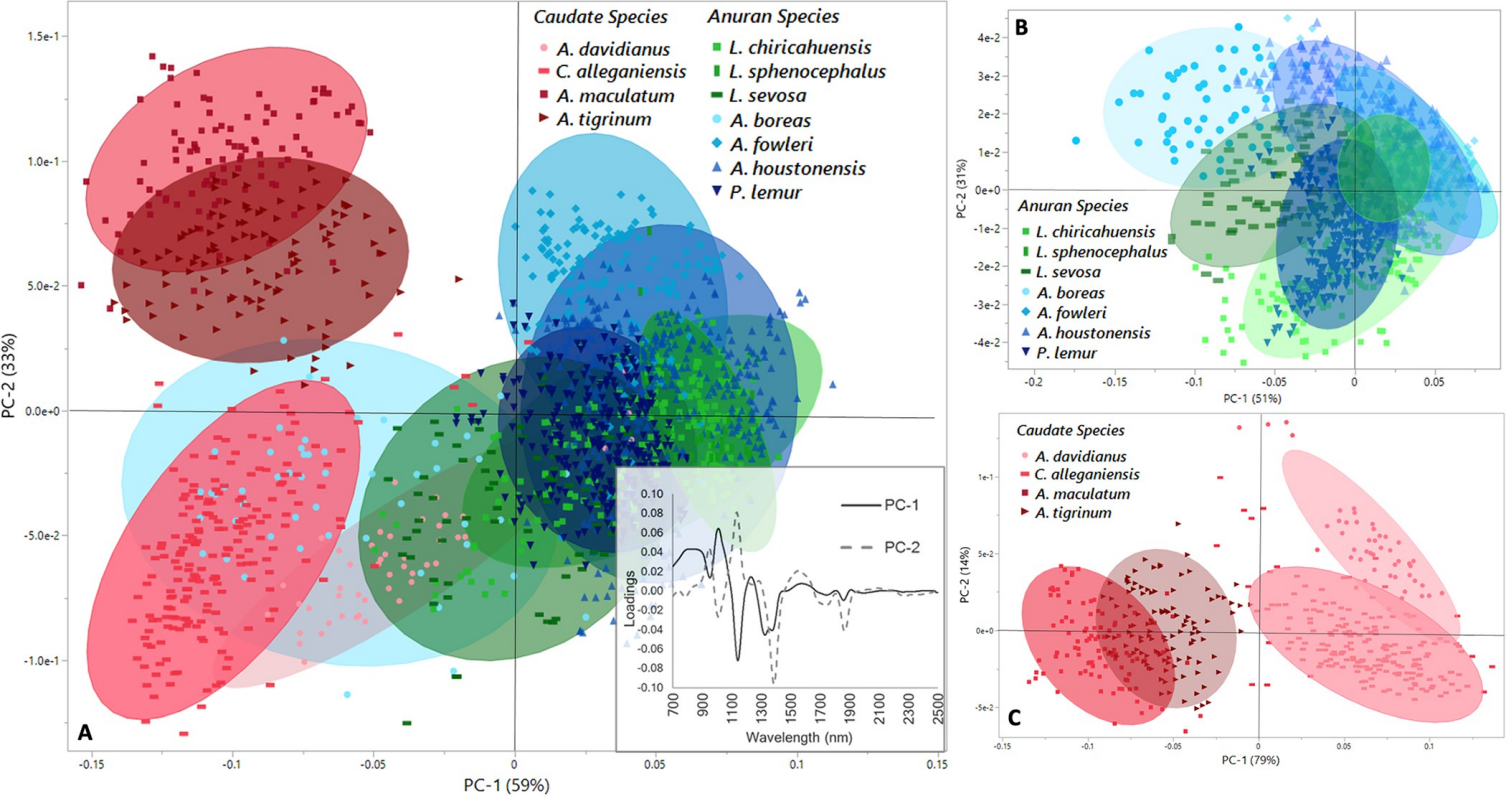
Principal component analysis scores plots for the transformed NIR spectra (700–2500 nm). A) PCA scores plot including all 11 species indicates distinct patterns between taxonomic orders, with greater spectral differences observed among species belonging to different orders than within orders. Inset: PCA loadings (700–2500 nm) highlighting the dominant peaks that explain the trends present in the scores plot: PC-1 (59%) and PC-2 (33%) explained 92% of database variance. B) PCA scores plot including only the seven anuran species. PC-1 (51%) and PC-2 (31%) explained 82% of the database variance. C) PCA scores plot including only the four caudate species indicate that greater biochemical differences occur between genera than within. PC-1 (79%) and PC-2 (14%) explained 93% of database variance.

**Fig 3:**
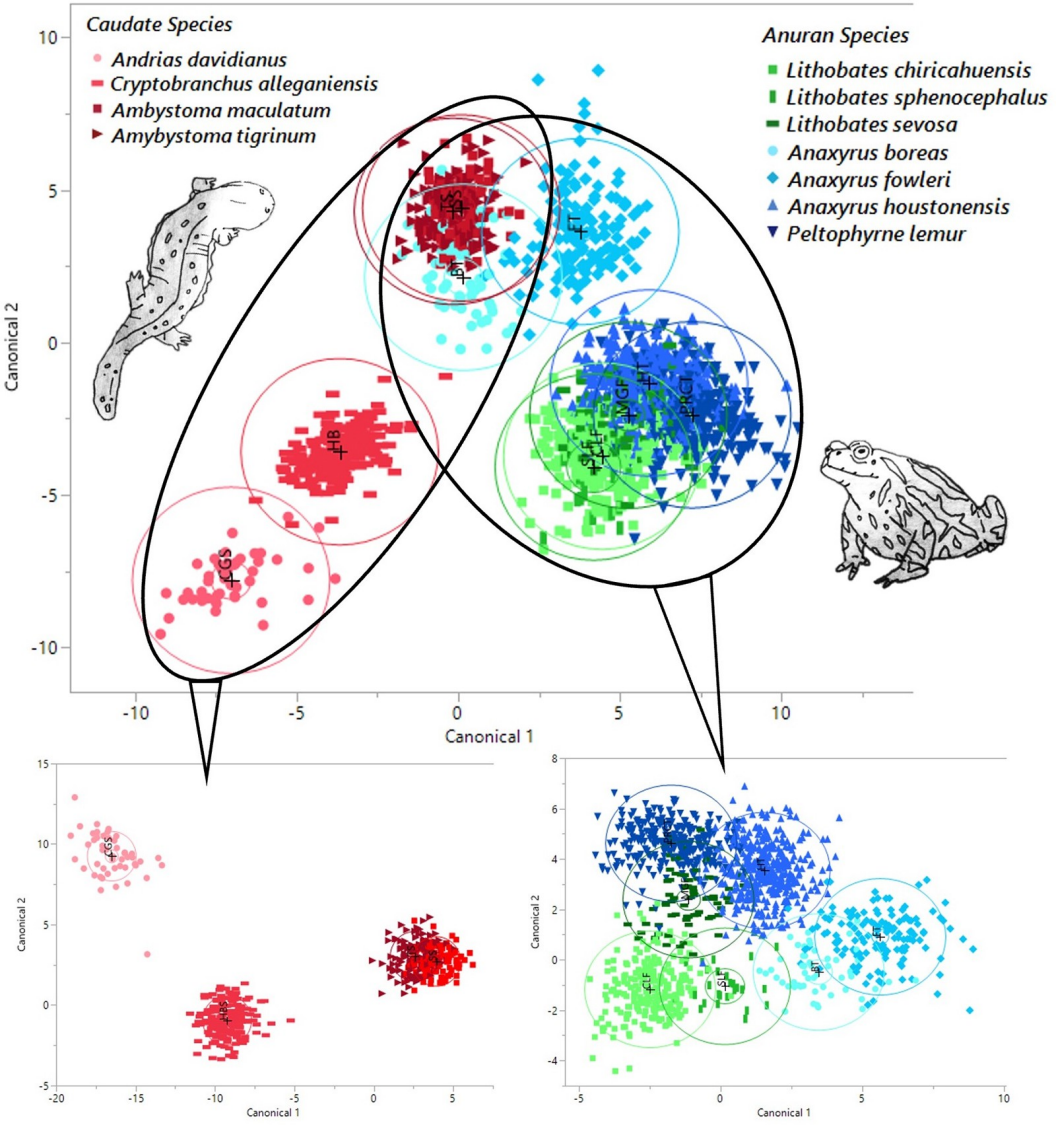
Canonical discriminant plots indicate biochemical separation between individuals belonging to different orders of amphibians (caudates located in the left oval and anurans located in the right oval). Discriminant plots visualizing biochemical relationships among the four caudates (bottom left) and seven anurans (bottom right) were also investigated. Illustrations by DMC.

The principal component scores plot is a representation of the original dataset that has been linearly transformed to a new coordinate-based system, such that the variables spanning 700–2500 nm are combined and reduced (i.e., based off their collinearity with one another) into linear combinations of latent variables called principal components (PC). PCs help to explain the total variance present in the dataset; the highest percentage of variance is explained by the first PC, then the second PC, and so on. Overall, the anuran (i.e., frogs and toads) spectral scores spanned all four quadrants of the PCA scores plot, whereas the majority of spectra belonging to caudates (i.e., salamanders) were strongly constrained to the negative axis of PC-1 (Fig 2). The biochemical similarities/differences inherent to the spectra are explained by the dominant peaks found in the positive and negative direction of the PCA loadings (Fig 2A inset), where the first two PCs accounted for 92% of the total variance present for the combined (anuran + caudate) database. Similarly, PC-1 and PC-2 accounted for 82% and 93% of the variance observed in the anuran-only and caudate-only databases, respectively. The loadings for both PC-1 and PC-2 varied distinctly at the following approximate wavelengths: 1100, 1400, and 1900 nm, as shown in Fig 2. The Hotelling’s T^2^ influence plot produced by the PCA indicated that no outliers were present in the spectral dataset. Furthermore, all anuran species were clustered in close proximity to each other, with the greatest spectral overlap occurring between species belonging to the same genera (e.g., *Anaxyrus* species) and the greatest separation occurring between genera (e.g., *Anaxyrus* vs. *Lithobates*, Figs 2B and 3). Similarly, all caudate species were clustered in close proximity to one another, with very tight clustering observed among individuals belonging to the same genera (e.g., *Ambystoma* species) and distinct spectral separation occurring between genera (e.g., *Ambystoma* vs. *Andrias* and *Cryptobranchus*, Figs 2C and 3). For example, spotted and tiger salamanders belonging to the family, Ambystomatidae, were closely clustered and constrained to the negative hemisphere of PC-1, while Chinese giant and hellbender salamanders belonging to the family, Cryptobranchidae, were tightly clustered within the positive hemisphere of PC-1.

### Model performance

**Fig 4:**
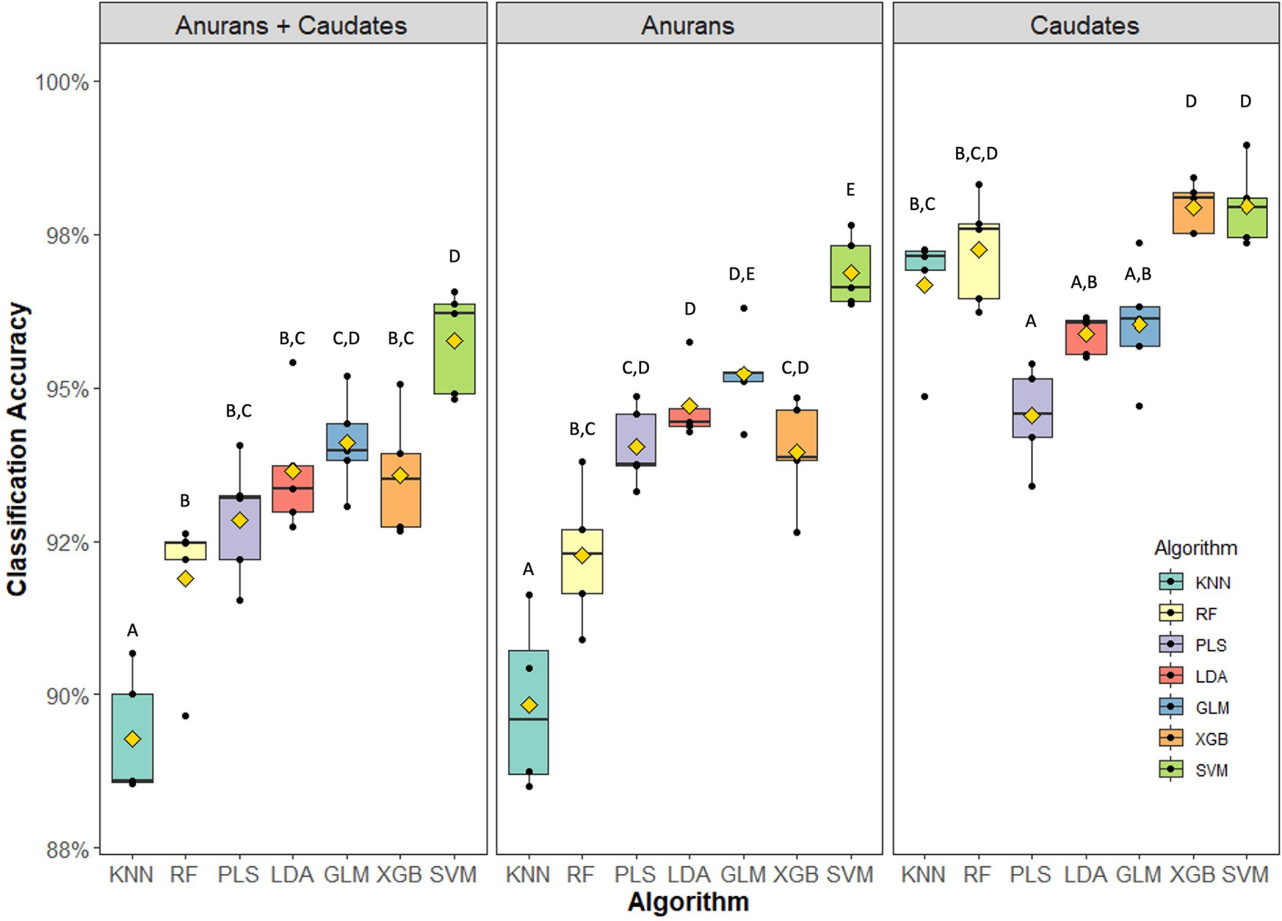
Comparison of mean classification accuracies between the seven models investigated. Models that differed significantly with regard to classification accuracy (*p* < 0.05; Tukey HSD) are assigned different letters. KNN = K-nearest neighbors, RF = random forest, PLS = partial least squares, LDA = linear discriminant analysis, GLM = generalized linear model with elastic net regularization, XGB = extreme gradient boosting, and SVM = support vector machine. Minima and maxima are indicated by whiskers, lower and upper interquartile ranges by boxes, medians by black horizontal lines, and means by gold diamonds.

When classifying spectra for the combined anurans and caudates dataset, we observed significant differences in mean classification accuracy across the seven models tested (*F*_6,98_ = 8.3, *p* < 0.05, ANOVA, Fig 4), for which the accuracy of the algorithms ranged from 89.3 ± 1.0% (KNN) to 95.8 ± 0.8% (SVM). In terms of mean misclassification error, KNN incorrectly classified 10.7 ± 1.0% of all observations, while the SVM misclassified only 4.2 ± 0.8% of the test dataset, indicating that the SVM made roughly two-fold fewer misclassifications than the KNN model. Additionally, the SVM model (95.8 ± 0.8%) yielded significantly higher classification accuracies compared to the RF (91.9 ± 1.2%), PLS (92.8 ± 0.9%), XGB (93.6 ± 0.9%), and LDA (93.6 ± 0.9%) models (*p* < 0.05, Tukey HSD), but no difference was found between the SVM (95.8 ± 0.8%) and GLM (94.1 ± 0.7%, *p >* 0.05) models. A table of performance results for each of the algorithms and datasets evaluated is shown in S1 Document.

When classifying spectra for the anuran-only dataset, a similar pattern was observed with regards to model performances, where KNN (89.3 ± 1.5%) yielded a significantly lower (*F*_6,28_ = 31.6, *p* < 0.05, Fig 4) mean classification accuracy than SVM (96.9 ± 0.5%). In addition, RF (92.3 ± 1.0%), PLS (94.0 ± 0.6%), LDA (94.7 ± 0.5%), GLM (95.2 ± 0.7%), and XGB (94.0 ± 0.8%) had moderate levels of model performance, relative to KNN and SVM (see Fig 4 for pairwise comparisons).

When classifying spectra for the caudate-only dataset, the PLS model (94.5 ± 0.7%) yielded a significantly lower (*F*_6,28_ = 13.1, *p* < 0.05) aggregated classification accuracy compared to KNN (96.7 ± 0.9%), RF (97.2 ± 0.8%), XGB (97.9 ± 0.4%), and SVM (98.0 ± 0.6%) models. PLS (94.5 ± 0.7%), LDA (95.9 ± 0.3%), and GLM (96.0 ± 0.9%) did not differ significantly (*p* > 0.05) with regard to mean classification accuracy (see Fig 4 for all other pairwise comparisons). Overall, mean classification accuracy was numerically greater for the caudates-only dataset (96.6 ± 0.2%) compared to the anurans-only (93.8 ± 0.3%) and combined (anurans + caudates; 93.0 ± 0.1%) datasets. The vast majority of the misclassified spectra occurred between species classifications that were more taxonomically related (Table 2). For example, species belonging to the anuran order tended to be misclassified as other anurans, with the exception of three boreal toad spectra that were misclassified as caudates (Table 2). Likewise, the majority of misclassified caudate spectra were assigned as similarly related caudate species.

**Table 2:** Confusion matrix of results for the support vector machine (i.e., the top-performing model) applied to the combined (anuran + caudate) dataset. Bolded values indicate the number of accurately classified spectra and non-bolded values indicate the misclassified spectra in the test dataset, respectively. Dotted lines separate major taxonomic orders (Anura vs. Caudata) and reveal that most spectra misclassifications occur within more closely related species.

	PREDICTION										
REFERENCE	CLF	SLF	DGF	BT	FT	HT	PRCT	CGS	HBS	SS	TS
**CLF**	**218**	1	0	0	0	3	2	0	0	0	0
**SLF**	0	**23**	0	0	0	0	0	0	0	0	0
**DGF**	4	0	**65**	0	0	0	4	0	0	0	0
**BT**	0	0	0	**50**	0	0	0	0	0	0	1
**FT**	0	0	0	0	**138**	0	0	0	0	0	1
**HT**	8	2	0	0	3	**391**	11	0	2	0	0
**PRCT**	1	0	4	0	0	5	**268**	0	1	0	0
**CGS**	0	0	0	0	0	0	0	**47**	0	0	0
**HBS**	0	0	0	1	0	0	0	1	**190**	0	0
**SS**	0	0	0	0	0	0	0	0	0	**96**	3
**TS**	0	0	0	2	0	0	0	0	1	3	**109**

Abbreviations: CLF = Chiricahua leopard frog, SLF = southern leopard frog, DGF = dusky gopher frog, BT = boreal toad, FT = Fowler’s toad, HT = Houston toad, PRCT = Puerto Rican crested toad, CGS = Chinese giant salamander, HBS = hellbender salamander, SS = spotted salamander, and TS = tiger salamander.

## Discussion

The current study investigated the use of a multi-model benchmark approach for evaluating amphibian taxonomic status using a spectroscopic dataset. Several key observations were made based off the results from the specific datasets utilized, the algorithms tested, and the input parameter settings (i.e., the tuned parameters and hyperparameters specific to each model) applied to achieve the classification task at hand. First, we provide support for near infrared spectroscopy coupled with chemometrics as an analytical technique for detecting spectral differences at the taxonomic levels of order, family, genus, and species level for the anurans and caudates examined. Robust prediction models were produced to accurately classify the eleven amphibian species in our study, and we observed greater spectral similarities among individuals within the same taxonomic group than between taxonomic groups. Second, the multi-algorithm approach applied here demonstrates the ability of various machine learning algorithms to model and ultimately predict the species of unknown individuals based off of their spectral profiles. As indicated by significantly higher classification accuracies for both SVM and GLM compared to LDA and KNN models, we conclude that different modeling techniques have the ability to perform predictive species assignments of spectra collected from live amphibians with varying levels of success. Thus, the use of a multi-model approach may be especially beneficial for evaluating new questions and datasets for the first time, as an algorithm that produces the best performance for one dataset or question of interest may not be suitable across differing study systems.

In this study, we demonstrate that the spectral profiles that vary between species can be readily discriminated from the dermal tissue of living individuals in a non-invasive manner, with minimal handling and without the use of anesthetics. Despite the high water content present in amphibian skin, all prediction models classified the eleven amphibian species with a high degree of accuracy (>89%). In the present study, the SVM yielded the highest mean classification accuracy and KNN the lowest among the seven algorithms tested. In another study utilizing a multi-model approach, Benson et al. [21] identified KNN as the top-performing model for discriminating fish species via spectral scanning of otoliths, which highlights the importance of testing multiple models when evaluating new datasets and different species or testing physiological parameters for the first time. These findings are in accordance with the “no free lunch” theorem [82], which states that no single model is guaranteed to outperform competing models for any given machine learning problem. While classic chemometric modeling methods have proven useful for generating prediction models, it would likely benefit researchers within the discipline of wildlife science to employ a multi-model benchmark approach when undertaking novel investigations. Multi-model benchmarks can be employed at low cost and with relative ease using open-source software analytics programs and the code provided (S2 Document). Additionally, as software updates are regularly released by chemometric analysis programs, utilization of this approach may be facilitated in future studies.

For the vast majority of the misclassified spectra in this study, inaccurate predictions occurred between species classifications that were more taxonomically related. For example, spectra from caudate species tended to be misclassified as other caudates, and a similar pattern was upheld among misclassified anuran spectra, with the exception of the boreal toad. We are unsure why three boreal toad spectra were classified as caudates, but it could be a result of boreal toads being a more cold-adapted species and experiencing similar environmental pressures faced by caudates [83,84]. The no-information rate, a metric that represents the best possible guess that can be made in the absence of information (i.e., the largest proportion of the dataset, or the most common class), was found to be 24%, suggesting that each of the models produced contained relevant information for predicting species designation in the current dataset (i.e., all models performed significantly better than a featureless, non-informative model) [4,85]. In the case of the 11-class classification problem (anurans + caudates combined), 24% corresponds to the proportion of spectra belonging to the majority class (*N = 399* spectra for *Anaxyrus houstonensis*) to the total number of spectra (*N* = 1661 spectra; Table 2).

Overall, the unique spectral profiles present between species likely represent a suite of biochemical differences in their skin that may be exploited for distinguishing individuals at the species level. We found that spectra could be used to group species based on taxonomic clade, as indicated by the principal component scores and canonical discriminant plots. For example, salamander spectra (i.e., Order Caudata) were clustered in close proximity to one another, while spectra belonging to frogs and toads (i.e., Order Anura) tended to remain spatially distinct from their caudate counterparts. The PCA loadings for caudates versus anurans indicated differences at 1100, 1400, and 1900 nm approximately; these wavelengths coincide with absorbance frequencies of biochemical functional groups indicating different distributions of biological compounds in the matrix of living tissue, which explain the trends presented in the scores plots.

The spectral profiles between species were then evaluated at a finer taxonomic scale by analyzing anurans and caudates separately. When evaluating anuran spectra only, spectra belonging to the frog genus, *Lithobates*, were very tightly clustered within genera yet were spatially distinct from spectra belonging to the toad genus, *Anaxyrus*. Altogether, these findings suggest that clear genetic distinctions among genera produce signature spectral profiles that may become more distinct as the genetic distance between species increases. This study is the first to our knowledge to demonstrate the use of NIR spectroscopy for both anuran and caudate taxonomic designations using live, whole animal spectroscopic data. Scanning of live animals in real-time provides a unique opportunity to assess the *in-vivo* status of an individual. On the other hand, spectral analysis of biological and excreta samples for making remote predictions of what was occurring in the animal before/at the time of sample collection is also useful for applications in wildlife management and conservation [1,2].

The biochemical phenotypes inherent to each species, that present as different spectral profiles, are likely because amphibian skin is a complex mucosal organ that performs various critical physiological functions, including respiration, water uptake, ion transport, and innate immunity, whose composition will differ between species [86–88]. Amphibian skin serves as a crucial immune organ constituting a sophisticated network of microbiological, physical, immunological, and chemical barriers to pathogen insult, as a result of having a wide range of secretory glands such as mucosal, granular, and adhesive glands [87]. The presence and function of these glands is influenced by the varying environmental pressures faced by species as well as evolutionary mechanisms which serve to aid species survival and reproduction. For example, frogs in the genus *Dermatonutus* produce specialized intraspecific chemicals that are excreted from breeding glands, similar to pheromones produced by the mental gland of plethodontid salamanders during the breeding season [86, 87]. Likewise, salamander skin has evolved crucial evolutionary mechanisms for purposes such as predator defense and mate attraction; *Plethodon shermani* has specialized granular glands on the dorsal side of their tail that excrete a toxic, sticky protein that deters predators, while the glands on the ventral side of the tail produce pheromones used for scent marking and courtship-related behaviors [89]. It is therefore crucial to consider the location of such glands when selecting the most appropriate sampling region for spectral data collection. The most optimal sampling region may very well differ depending on the physiological trait being modeled.

With regard to species investigated in the current study, adult hellbender salamanders rely solely on cutaneous breathing, making the skin an important site of electrolyte and gas exchange [90]. Additionally, the skin of the Chinese giant salamander was found to contain 249 unique proteins, 155 of which are derived from the skin mucous alone [91]. Members of the *Anaxyrus* genus possess granular glands that secrete peptides, biogenic amines, steroids, and alkaloids, which serve anti-predatory functions [92]. Furthermore, within the *Anaxyrus* genus alone, there is a tremendous amount of amine and polypeptide diversity in the skin both inter-specifically and intra-specifically [93], which may have contributed to the unique spectral profiles observed among anurans in the present study. Amphibian skin also serves as an osmoregulatory organ that regulates acid-base balances in the body and the movement of hydrominerals [88]. The epithelial tissue contains vital sodium, urea, and water channels (i.e., aquaporins) that are constantly changing factors affecting skin biochemistry [88]. Given that different amphibian species produce varying types and amounts of glycoproteins and antimicrobial peptides, the types of fungal and bacterial communities that are harbored on the skin (i.e., microbiome) of individual species may also differ [94]. Altogether, the enormous amount and phenotypic plasticity of biochemical diversity present within amphibian skin likely explains the high performance of near infrared spectroscopy for identifying various traits.

Multi-model benchmarks and ensembles have been explored and used extensively across several industries. Such an approach has become commonplace in fields such as engineering, medicine, and agriculture [4–8,10–12,14,15,95]. Here, the application of a multi-model approach for optimizing spectroscopic prediction models in wildlife science is demonstrated. Considering the multivariate nature of large spectroscopic datasets, machine learning methodologies are well suited to address questions in wildlife science. Of importance to note is that ensembles may refer to at least two different modeling concepts. The first is similar to the multi-model approach employed in the current study, where numerous algorithms are applied to address the classification or regression problem on the same dataset, except results are combined in concert to base predictions. Ensembles go a step further in that the model acknowledges that one algorithm may perform more effectively for predicting a specific class, relative to other algorithms tested; for example, one algorithm may more effectively predict the majority class present in the dataset, while another may classify the minority class at a higher rate. On their own, it may be that neither algorithm is useful for predicting both classes, but when the algorithms are combined in an ensemble, they can achieve higher prediction rates than if they were operated separately [6]. The concept of ensembling is also similar to stacking, where models are stacked on top of one another to base decisions, which may ultimately lead to the generation of a model with greater predictive performance. Alternatively, the term “ensemble” could refer to algorithms such as random forest, a model which builds numerous independent trees; each tree gets their own “vote”, which is then tallied up in an ensemble to make the final prediction [96]. In a random forest, the voting weight is equal amongst all trees, whereas for extreme gradient boosting algorithm, the vote is weighted by the overall accuracy yielded by a tree during the model calibration stage of model development. Furthermore, varying procedural steps used by each unique algorithm to make predictions likely contribute toward differences in their respective classification performance [4]. As similar benchmarking approaches are applied across a wider range of species and tissue types (e.g., fin clip, vertebra, hair, blood, feces) in the future, candidate algorithm(s) most appropriate for specific study paradigms may be identified.

Based off the results from this study, we propose the application of a multi-model benchmark approach to evaluate the effectiveness of multiple algorithms for producing the best performance results. The algorithm that may perform best on one spectral dataset for any given classification or regression problem may differ depending on the problem and target species or sample matrix being investigated. Overall, this study demonstrates the utility of a multi-model benchmark approach for comparing and optimizing model performance using seven conventional machine learning algorithms. With that being said, other modeling frameworks, such as deep learning (e.g., convolutional neural networks) have been recently established and may serve to increase the predictive capacity of spectroscopic models built for addressing questions in wildlife science.

### Dealing with imbalanced datasets

Imbalanced sample sizes among treatment groups are a major yet common obstacle to producing robust prediction models, especially in field settings where study populations are often limited. To overcome challenges concerning imbalanced datasets (e.g., when a class is either over- or under-represented), sample equalization can be conducted using a variety of methods, including under-sampling, over-sampling, augmenting the weight of the minority class, and through the synthetic minority over-sampling technique (SMOTE) [4]. SMOTE helps to balance the samples for each class by generating new samples for the minority class; this procedure can be used in conjunction with other sample equalization techniques (e.g., under-sampling of the majority class). However, it is important to note that although over-sampling the minority class or under-sampling the majority class can help to improve model performance measures for the newly balanced class, it may in turn negatively impact model performance for predicting other classes [97]. For the present study, we acknowledge the imbalance of spectra between the eleven species investigated, with the greatest disparity occurring between the southern leopard frog (*N* = 26 spectra) and Houston toad (*N* = 399 spectra). This represents the classic problem of imbalanced datasets where the minority class (i.e., southern leopard frog in this case) is consequently the most misassigned class in the prediction model (Table 2). In such a case, the use of a class weighting procedure might be considered, which assigns a higher penalization value for misclassifying the minority class. Although an important consideration, methods accounting for class imbalance were not relevant to the objectives of this study and were thus not conducted.

### Feature selection

Feature selection is a critical data processing step for exploring the dataset and evaluating the usefulness of the predictor variables, which is especially important for large spectroscopic datasets containing thousands of variables. Given that there are 1800 wavelengths in the NIR spectrum (700–2500 nm), each serving as a potential variable to predict the response, numerous methods have been employed to reduce database dimensionality in an effort to parse out meaningful variables in a sea of non-informative features. The variable reduction technique (also referred to as feature engineering) used here was Boruta (see Methods), although many other tools are available for reducing database dimensionality. Principal component analysis (PCA) is by far the most widely used method in spectroscopy studies, followed by three commonly used methods for reducing database complexity. First, some algorithms, such as linear discriminant analysis (LDA), partial least squares (PLS), and random forest (RF) internally perform “dimensionality reduction” processes. Second, “filter methods” may be used to compare the features (i.e., wavelengths) to one another and the outcome to determine relative importance of the features for predicting the response [98]. For example, there are many filters available in the “mlr” package within R, all of which can be used to rank features based on their importance for the desired task. More specifically, filters such as the ‘ranger_permutation’ function works by dropping features from the dataset and observing how well the model performs in the absence of the excluded feature. Third, “wrapper” methods can be used and are similar to filter methods but make decisions on feature importance based on the model being built [99]. For example, a sequential model wrapper may be used to select the optimal number of features by employing either forward or backward selection, which adds on or removes features sequentially at each step, similarly to stepwise regression, which only retains the most informative factors [99]. As opposed to feature selection using filters, wrapper techniques use the model itself and thus wrapper models may yield more accurate results that come at the cost of having significantly longer run times during the calibration process. Given that both the quantity and quality (i.e., the informative value) of selected variables may vary between methods, ample consideration should be given to feature selection procedures during the model development stage. Variable selection methods for NIR spectroscopy have been extensively described in a recent work by Yun et al. [98].

### Cross-validation schemes

Cross-validation is crucial for addressing concerns related to model overfitting and helps to avoid over-optimistic performance results for the training and test datasets [4]. A recent study by Au et al. [100] employed a variety of cross-validation schemes (i.e., leave one out, leave group out, and random subset k-fold) to evaluate calibration and predictive error for assessing chemical compounds in eucalyptus leaves. They found that the method in which spectral observations were split (i.e., for calibration, cross-validation, and testing datasets) was important for fitting generalizable prediction models while minimizing error and bias for the cross-validation and prediction datasets. Additionally, Au et al. [100] observed decreases in root mean square error prediction when the number of calibration/training samples increased, while prediction error plateaued when the calibration dataset reached a sufficient sample size.

The current study used a five-fold nested resampling scheme [101,102], which was selected after testing a variety of resampling methods in a pilot study. We observed that mean classification accuracies were highest and error minimized to the fullest extent when cross-validation was applied to both the inner (i.e., used for model calibration and tuning) and outer (i.e., used for testing) sampling datasets, whereas accuracy was lowest and variance the highest when holdout methods were solely applied to the outer resampling. Thus, testing and comparing various cross-validation schemes simultaneously may help to identify which cross-validation procedures provide the least unbiased estimates of predictive performance while minimizing error [102]. Further considerations of cross-validation procedures for producing robust, broad-based models are outlined in detail by Au et al. and Cozzolino [100,103]. Although the nested cross-validation applied in this study is designed to provide a reliable estimate of model performance, a separate external dataset could serve as a final validation step to assess model efficacy on a completely unseen test dataset, which may be a valuable consideration for future studies.

### Other algorithms, parameter, & hyperparameter tuning

Within freely available, open-source statistical programs such as R, it is possible to access numerous algorithms for model development and testing, each with their own unique set of tunable parameters and/or hyperparameters. During the calibration stage where models are tuned to achieve peak performance for the measure of interest (e.g., accuracy, mean misclassification error, kappa, ROC, AUC), tuning wrappers are employed with set search spaces, which can be conducted either at random with a given set of parameters or systematically in a grid-like fashion. While some algorithms, such as LDA or PLS have only one tunable parameter (i.e., number of principal components/factors applied), others such as GLM and XGB may have numerous tunable hyperparameters. Changing the search method as well as increasing or decreasing both the search space and the hyperparameters may greatly affect both the overall performance and run time for the prediction model. The cross-validation procedure that is applied can also profoundly influence the time required to calibrate the model (e.g., nested cross-validation is more computationally intensive than conventional k-fold cross-validation) [101]. At any rate, after the prediction model has been adequately calibrated and tested, the tuned model can be used to make predictions at much faster rates.

## Materials and methods

### Ethics statement

Experiments were approved by and adhered to the permit and/or animal care and use guidelines set forth by the Fort Worth Zoo (IACUC #17-H001; Federal Permit TE051818-0), Ladder Ranch (USFWS Permit #TE43754A-0), and Mississippi State University (IACUC #19–345).

### Study population & data acquisition

**Fig 5:**
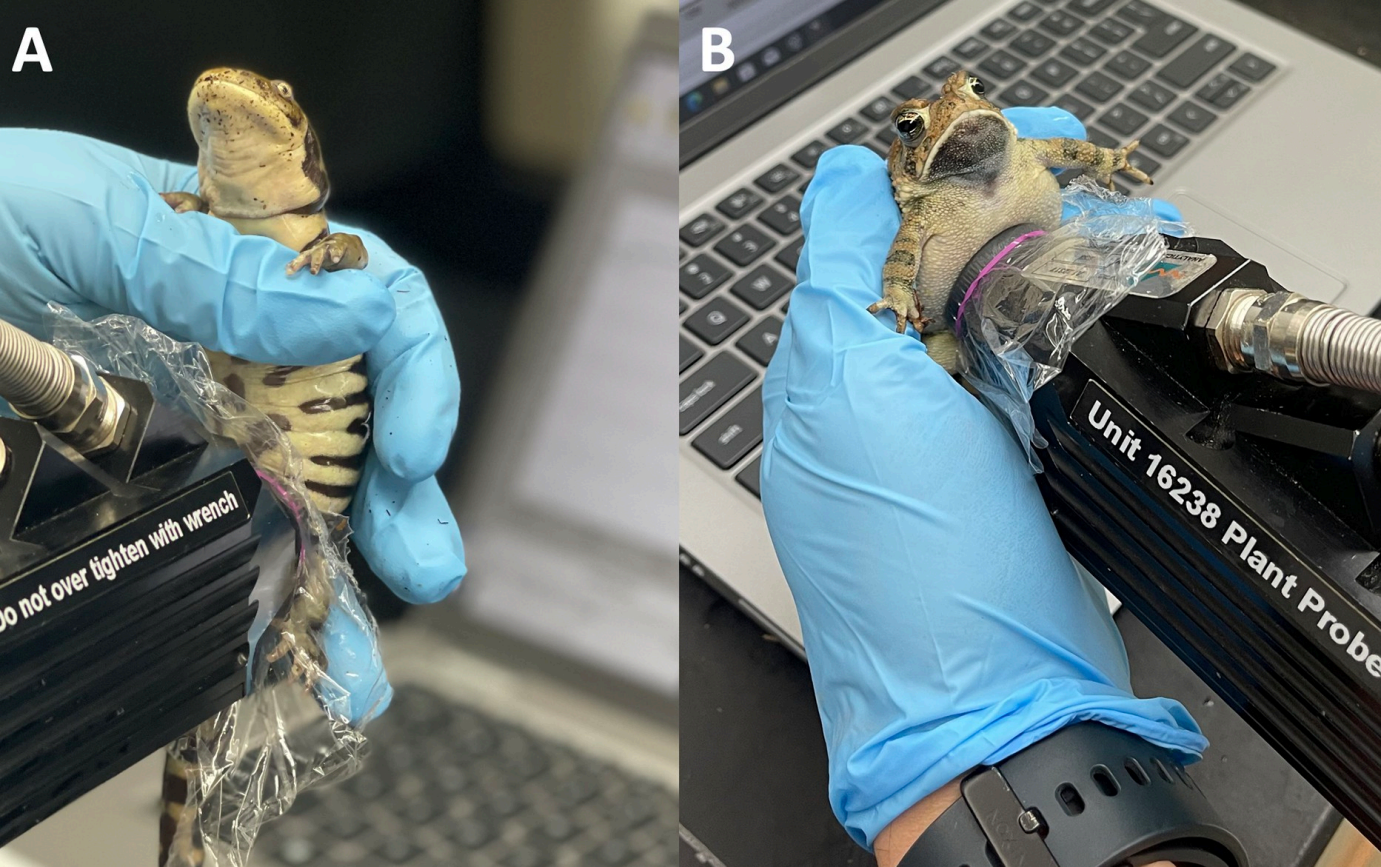
**Examples of NIR spectra acquisition from live A) tiger salamander (*Ambystoma tigrinum*) and B) Fowler’s toad (*Anaxyrus fowleri*).** Spectra were non-invasively collected superior to the cloaca on the ventral side for all 11 of the amphibian species investigated. Photos taken by DMC.

NIR reflectance spectra (*N* = 1661) were collected with an ASD FieldSpec 3 IndicoPro (Malvern Panalytical, ASD Analytical Spectral Devices Inc., Boulder, CO, USA). Spectra were collected using a 2 cm diameter low-powered plant contact probe (as per [19]; Fig 5) from the cloacal region of eleven amphibian species, seven of which were anuran species (frogs or toads) including the boreal toad (*Anaxyrus boreas*, *N =* 53 individuals), Fowler’s toad (*Anaxyrus fowleri*, *N =* 47), Houston toad (*Anaxyrus houstonensis*, *N* = 133), Puerto Rican crested toad (*Peltophryne lemur*, *N* = 95), Chiricahua leopard frog (*Lithobates chiricahuensis*, *N* = 77), southern leopard frog (*Lithobates sphenocephalus*, *N =* 26), and dusky gopher frog (*Lithobates sevosa*, *N* = 69), and the remaining four species were caudates (salamanders) including the spotted salamander (*Ambystoma maculatum*, *N* = 33), tiger salamander (*Ambystoma tigrinum*, *N* = 38), Chinese giant salamander (*Andrias davidianus*, *N* = 48), and the eastern hellbender salamander (*Cryptobranchus alleganiensis*, *N =* 49). All spectra were collected across the wavelength range of 350–2500 nm (resolution of 1.4 nm for the 350–1000 nm range and 2 nm for the 1000–2500 nm range), where each spectral replicate was comprised of 50 scans then averaged into a single spectrum (integration time approximately 34 ms). Data were analyzed using three separate datasets: (1) anurans and caudates combined (*n* = 11 species); (2) anurans only (*n* = 7 species); and (3) caudates only (*n* = 4 species). Spectra were collected opportunistically as part of collaborative studies conducted at the Fort Worth Zoo (Fort Worth, TX), Turner Enterprise’s Ladder Ranch (Caballo, NM), and Mississippi State University’s Conservation Physiology Lab (Mississippi State, MS).

### Data preprocessing & feature selection

Chemometric analyses were carried out in R version 4.1.2 [104] and R Studio version 2020.02.0 [105] using the “mlr” package [106]. The packages “xgboost” and “glmnet” were used to access the extreme gradient boosting and GLM algorithms, respectively [107,108]. Regions within the visible-light spectrum were omitted so that only wavelengths within the near infrared range (700–2500 nm) would be included as predictor variables (often referred to as “features” in machine learning) in our analysis. Species class was used as the response variable (also referred to as the “task”). The task for the model also includes information to specify predictor variables, if a blocking procedure is to be used, and whether the task is a classification or regression problem. Spectral data were preprocessed with smoothing (Savitsky-Golay, first order polynomial with a window size of 11) and first derivative (Savitsky-Golay, first order polynomial with 3 symmetric kernel points) functions using the “prospectr” package [109]. The “Boruta” package was applied as the variable selection algorithm [110]. The Boruta algorithm functions by using feature importance scores assigned to each original feature that competes against shadow features, which are latent variables representative of the original variables that are randomized to a new permutated form; only variables that score higher than the best shadow feature in the set for predicting the response variable in at least 95% of trials are selected as “important” [110]. This significantly reduced the total number of variables in the dataset from 1800 to 906. Parameters “ntrees” and “maxRuns” affect the number of trees employed in the random forest algorithm used within Boruta, and the maximum number of runs completed, respectively; modulating these parameters may affect the total number of features selected. The newly reduced dataset of 906 variables (i.e., wavelengths) was used to calibrate each of the prediction models for the combined-species dataset. As for the caudate-only and anuran-only datasets, 387 and 715 variables, respectively, were retained by Boruta to build prediction models.

### Cross-validation & model calibration

To avoid data leakage from the training dataset into the test dataset, we partitioned the full dataset into blocks and applied a five-fold cross-validation procedure. Due to several of the amphibian species being sampled with varying numbers of spectral replicates, the full dataset was partitioned into three blocks, depending on the number of spectral replicates collected for each species (range = 1–4 replicate scans; e.g., *A*. *davidianus* individuals had only one replicate taken versus three replicates taken for each *A*. *houstonensis* individual). The blocking procedure ensures that all replicate scans of an individual end up together in either the test or training dataset and removes the opportunity for these related scans to leak data between training and test datasets. The number of individuals, spectral replicates, and total number of spectra acquired for each species are provided in S3 Document. We used a nested resampling scheme where five-fold cross-validation was conducted on both the inner and outer resampling datasets; the inner resampling is used for model calibration and parameter tuning/optimization and the outer resampling returns results on the test dataset [101,111]. This cross-validation procedure is expected to reduce overfitting and the overall variance yielded in the estimated performance accuracies, as the resulting output is an aggregated performance measurement that is generated by taking an average of the five training runs (as opposed to a single training run) for five iterations [102]. More specifically, the outer sampling, comprised of the entire dataset, was split into five folds such that one fold was treated as the test dataset and the remaining four folds as the training dataset. The four latter folds were used in the inner resampling scheme and then followed a conventional five-fold cross-validation approach where hyperparameters were tuned for each iteration, and the hyperparameters yielding the highest aggregated results for the inner fold were then used for the prediction model’s outer fold of test data that was initially held out [101]. This process was run for five iterations (k = 5) so that each fold had a turn to be treated as test data; this is done by sequentially rotating test and training folds until each fold is represented in the test dataset yet remains independent from the training dataset. The five accuracies generated for each model were aggregated then compared using an ANOVA, and a Tukey HSD test (alpha = 0.05) was utilized to evaluate significant differences between the mean classification accuracies of all algorithms investigated. Assumptions for ANOVA were met according to Shapiro-Wilk, Bartlett’s, and Durbin-Watson tests. Canonical discriminant plots to visualize spectral separation between taxonomic classifications were generated using JMP 14.0 [112]. Variation between species designations was visualized by two canonical variables that are themselves uncorrelated with one another but each of which maximizes the correlations present for the species classification variable.

The ‘benchmark’ function in “mlr” was used to train and test each of the models using identical data splits for all seven algorithms to ensure that all models were directly comparable [106]. The ‘confusionMatrix’ function in the “caret” package was used to provide an output of performance metrics including accuracy, no-information rate, and confusion matrices [85]. Parameters and hyperparameters along with their tuning constraints (i.e., upper and lower range) for each of the algorithms applied are specified and briefly described in S4 Document. Confusion matrices for both the worst- and top-performing models for each of the three datasets (anurans + caudates combined, anurans-only, caudates-only) analyzed are reported in S5 Document. A literature search including research articles, published conference abstracts, and reviews was conducted in Google Scholar, Scopus, and Web of Science to determine the number of algorithms applied and tissue type sampled in each study (search words: “wildlife” AND “spectroscopy”). We recognize the search conducted is not exhaustive but consider it representative of the general modeling approach currently utilized for spectroscopy studies in wildlife science. We acknowledge the possibility that authors explored but did not explicitly report the use of additional algorithms tested during the exploratory stage of data analysis. In any case, we categorized studies as a single or multi-model (two or more) approach according to the number of algorithms reportedly used for tuning models and making predictions. Results presented in Table 1 include studies containing vertebrate species exclusively, and studies found in our literature search were excluded if they did not clearly specify the algorithm or model type used for generating prediction models. Additionally, our search was limited to studies utilizing shallow machine learning algorithms, although deep learning is an emerging predictive modeling technique in spectroscopic studies [80,113–115].

## Supporting information

S1 Document Table of performance results for each of the algorithms and datasets evaluated. (XLSX)

S2 Document R script for running a multi-model predictive modeling approach in R Studio. (RMD)

S3 Document Table indicating the number of individuals and sample replicates for each species examined in the study. (XLSX)

S4 Document Brief explanation of the algorithms applied in the manuscript and their respective hyperparameter tuning constraints. (DOCX)

S5 Document Confusion matrices for predicting taxonomic classifications for the anuran-only and caudate-only datasets. (XLSX)

S6 Document Spectral database for the eleven amphibian species examined in this study. (XLSX)

## References

[1] Vance CK, Tolleson DR, Kinoshita K, Rodriguez J, Foley WJ. Near infrared spectroscopy in wildlife and biodiversity. J Near Infrared Spectrosc. 2016;24(1):1–25.

[2] Morgan LR, Marsh KJ, Tolleson DR, Youngentob KN. The Application of NIRS to Determine Animal Physiological Traits for Wildlife Management and Conservation. 2021;1–14.

[3] Helser TE, Benson IM, Barnett BK. Proceedings of the research workshop on the rapid estimation of fish age using Fourier Transform Near Infrared Spectroscopy (FT-NIRS). AFSC Processed Report. 2019;53.

[4] Kuhn M, Johnson K. Applied Predictive Modeling. Springer; 2013.

[5] Yadav SS, Kadam VJ, Jadhav SM, Jagtap S, Pathak PR. Machine learning based malaria prediction using clinical findings. In: 2021 International Conference on Emerging Smart Computing and Informatics, ESCI 2021. Institute of Electrical and Electronics Engineers Inc.; 2021; p. 216–22.

[6] Mugo R, Saitoh SI. Ensemble Modelling of Skipjack Tuna (Katsuwonus pelamis) Habitats in the Western North Pacific Using Satellite Remotely Sensed Data; a Comparative Analysis Using Machine-Learning Models. Remote Sens (Basel). 2020;12(16):2591.

[7] Shahbaz M, Ali S, Guergachi A, Niazi A, Umer A. Classification of Alzheimer’s disease using machine learning techniques. In: DATA 2019—Proceedings of the 8th International Conference on Data Science, Technology and Applications. SciTePress; 2019; p. 296–303.

[8] Song H, Dong C, Zhang X, Wu W, Chen C, Ma B, et al. Rapid identification of papillary thyroid carcinoma and papillary microcarcinoma based on serum Raman spectroscopy combined with machine learning models. Photodiagnosis Photodyn Ther. 2022;37. doi: 10.1016/j.pdpdt.2021.102647 34818598

[9] Peppes N, Daskalakis E, Alexakis T, Adamopoulou E, Demestichas K. Performance of machine learning-based multi-model voting ensemble methods for network threat detection in agriculture 4.0. Sensors. 2021;21(22).7475. doi: 10.3390/s21227475 34833551 PMC8622709

[10] Khamparia A, Singh A, Anand D, Gupta D, Khanna A, Arun Kumar N, et al. A novel deep learning-based multi-model ensemble method for the prediction of neuromuscular disorders. Neural Comput Appl. 2020;32(15):11083–95.

[11] Xiao Y, Wu J, Lin Z, Zhao X. A deep learning-based multi-model ensemble method for cancer prediction. Comput Methods Programs Biomed. 2018;153:1–9. doi: 10.1016/j.cmpb.2017.09.005 29157442

[12] De Oliveira H, Prodel M, Augusto V. Binary Classification on French Hospital Data: Benchmark of 7 Machine Learning Algorithms. In: Proceedings—2018 IEEE International Conference on Systems, Man, and Cybernetics, SMC 2018. Institute of Electrical and Electronics Engineers Inc.; 2019. p. 1743–8.

[13] Mostafa FB, Hasan E. Machine Learning Approaches for Inferring Liver Diseases and Detecting Blood Donors from Medical Diagnosis. 2021. ArXiv, abs/2104.12055.

[14] Yeh C, Meng C, Wang S, Driscoll A, Rozi E, Liu P, et al. SustainBench: Benchmarks for Monitoring the Sustainable Development Goals with Machine Learning. 2021; Available from: http://arxiv.org/abs/2111.04724

[15] Brillante L, Bois B, Mathieu O, Lévêque J. Electrical imaging of soil water availability to grapevine: a benchmark experiment of several machine-learning techniques. Precis Agric. 2016;17(6):637–58.

[16] Cunha CL, Torres AR, Luna AS. Multivariate regression models obtained from near-infrared spectroscopy data for prediction of the physical properties of biodiesel and its blends. Fuel. 2020;261.

[17] Clavaud M, Roggo Y, Dégardin K, Sacré PY, Hubert P, Ziemons E. Global regression model for moisture content determination using near-infrared spectroscopy. European Journal of Pharmaceutics and Biopharmaceutics. 2017;119:343–52. doi: 10.1016/j.ejpb.2017.07.007 28729179

[18] Boucher TF, Ozanne M V., Carmosino ML, Dyar MD, Mahadevan S, Breves EA, et al. A study of machine learning regression methods for major elemental analysis of rocks using laser-induced breakdown spectroscopy. Spectrochim Acta Part B At Spectrosc. 2015;107:1–10.

[19] Chen LD, Santos-Rivera M, Burger IJ, Kouba AJ, Barber DM, Vance CK. Near-Infrared Spectroscopy (NIRS) as a Method for Biological Sex Discrimination in the Endangered Houston Toad (Anaxyrus houstonensis). Methods Protoc. 2021;5(1):4. doi: 10.3390/mps5010004 35076558 PMC8788558

[20] Steyaert SMJG, Hütter FJ, Elfström M, Zedrosser A, Hackländer K, Lê MH, et al. Faecal spectroscopy: A practical tool to assess diet quality in an opportunistic omnivore. Wildlife Biol. 2012;18(4):431–8.

[21] Benson IM, Barnett BK, Helser TE. Classification of fish species from different ecosystems using the near infrared diffuse reflectance spectra of otoliths. J Near Infrared Spectrosc. 2020;28(4):224–35.

[22] Wright C, Wedding BB, Grauf S, Whybird OJ. Age estimation of barramundi (Lates calcarifer) over multiple seasons from the southern Gulf of Carpentaria using FT-NIR spectroscopy. Mar Freshw Res. 2021;72(9):1268–79.

[23] Fuller K. Exploring Effects of Sample Storage, Preparation, and Tissue Type on Fourier Transform-Near Infrared Spectroscopy (FT-NIRS) Ageing across Fish Taxa. 2021. https://scholarcommons.sc.edu/etd/6227

[24] TenBrink T, Neidetcher S, Arrington M, Benson I, Conrath C, Helser T. Fourier transform near infrared spectroscopy as a tool to predict spawning status in Alaskan fishes with variable reproductive strategies. J Near Infrared Spectrosc. 2022;30(4):179–188.

[25] Helser TE, Benson I, Erickson J, Healy J, Kastelle C, Short JA. A transformative approach to ageing fish otoliths using fourier transform near infrared spectroscopy: A case study of eastern bering sea walleye pollock (gadus chalcogrammus). Can of Fish and Aqu Sci. 2019;76(5):780–9.

[26] Ishigaki M, Kawasaki S, Ishikawa D, Ozaki Y. Near-Infrared Spectroscopy and Imaging Studies of Fertilized Fish Eggs: In Vivo Monitoring of Egg Growth at the Molecular Level. Sci Rep. 2016 Jan 28;6. 10.1038/srep20066 PMC4730184 26818027

[27] Wedding BB, Forrest AJ, Wright C, Grauf S, Exley P, Poole SE. A novel method for the age estimation of Saddletail snapper (Lutjanus malabaricus) using Fourier Transform-near infrared (FT-NIR) spectroscopy. Mar Freshw Res. 2014;65(10):894–900.

[28] Passerotti MS, Helser TE, Benson IM, Barnett BK, Ballenger JC, Bubley WJ, et al. Age estimation of red snapper (Lutjanus campechanus) using FT-NIR spectroscopy: Feasibility of application to production ageing for management. ICES J of Marine Sci. 2020;77(6):2144–56.

[29] Rigby CL, Wedding BB, Grauf S, Simpfendorfer CA. A novel use of near infrared spectroscopy: ageing deep water sharks. NIR news. 2015;26(4):4–5.

[30] Rigby CL, Wedding BB, Grauf S, Simpfendorfer CA. The utility of near infrared spectroscopy for age estimation of deepwater sharks. Deep Sea Res 1 Oceanogr Res Pap. 2014;94:184–94. Available from: doi: 10.1016/j.dsr.2014.09.004

[31] Servid SA, Talbott MJ, Van Eenennaam JP, Doroshov SI, Struffenegger P, Webb MAH, et al. Rapid noninvasive characterization of ovarian follicular atresia in cultured white sturgeon (Acipenser transmontanus) by near infrared spectroscopy. Aquaculture. 2011;315(3–4):290–7.

[32] Arrington MB, Helser TE, Benson IM, Essington TE, Matta ME, Punt AE. Rapid age estimation of longnose skate (Raja rhina) vertebrae using near-infrared spectroscopy. Mar Freshw Res. 2021;73(1):71–80.

[33] Vance CK, Kouba AJ, Willard ST. Near Infrared Spectroscopy Applications in Amphibian Ecology and Conservation: Gender and Species Identification. NIR news. 2014;25(4):10–5.

[34] Torralvo K, Magnusson WE, Durgante F. Effectiveness of Fourier transform near-infrared spectroscopy spectra for species identification of anurans fixed in formaldehyde and conserved in alcohol: A new tool for integrative taxonomy. J of Zool System and Evol Res. 2021;59(2):442–58.

[35] Torralvo K, Durgante F, Pasquini C, Magnusson WE. Near infrared spectroscopy for the identification of live anurans: Towards rapid and automated identification of species in the field. J Near Infrared Spectrosc. 2023;31(2):80–88.

[36] Vance CK, Graham K, Kouba A, Swillard S. In vivo sex identification of the endangered Mississippi Gopher frog (Lithobates sevosa) using near infrared reflectance spectroscopy. 17th International Conference on Near Infrared Spectroscopy. 2015; #34396.

[37] Vance CK, Kouba AJ, Zhang HX, Zhao H, Wang Q, Willard ST. Near Infrared Reflectance Spectroscopy Studies of Chinese Giant Salamanders in Aquaculture Production. NIR news. 2015;26(2):4–7.

[38] André J, Gyuris E, Lawler IR. Comparison of the diets of sympatric dugongs and green turtles on the Orman Reefs, Torres Strait, Australia. Wildlife Res. 2005;32(1):53–62.

[39] Cornejo J, Taylor R, Sliffe T, Bailey CA, Brightsmith DJ. Prediction of the nutritional composition of the crop contents of free-living scarlet macaw chicks by near-infrared reflectance spectroscopy. Wildlife Res. 2012;39(3):230–3.

[40] Landau S, Nitzan R, Barkai D, Dvash L. Excretal Near Infrared Reflectance Spectrometry to monitor the nutrient content of diets of grazing young ostriches (Struthio camelus). S Afr J Anim Sci. 2006;36(4):248–56.

[41] Williams CL, Meir JU, Ponganis PJ. What triggers the aerobic dive limit? Patterns of muscle oxygen depletion during dives of emperor penguins. J of Exp Bio. 2011;214(11):1802–12. doi: 10.1242/jeb.052233 21562166 PMC3092726

[42] Liu X, Sun J, Sun L, Liu W, Wang Y. Reflectance spectroscopy: A new approach for reconstructing penguin population size from Antarctic ornithogenic sediments. J Paleolimnol. 2011;45(2):213–22.

[43] Sheng Q, Santos-Rivera M, Ouyang X, Kouba AJ, Vance CK. Near-Infrared Spectroscopy and Mode Cloning (NIR-MC) for In-Situ Analysis of Crude Protein in Bamboo. Remote Sens (Basel). 2022;14(6):1302.

[44] Wiedower EE, Kouba AJ, Vance CK, Hansen RL, Stuth JW, Tolleson DR. Fecal near infrared spectroscopy to discriminate physiological status in giant pandas. PLoS One. 2012;7(6). doi: 10.1371/journal.pone.0038908 22719982 PMC3374779

[45] Kinoshita K, Miyazaki M, Morita H, Vassileva M, Tang C, Li D, et al. Spectral pattern of urinary water as a biomarker of estrus in the giant panda. Sci Rep. 2012;2. doi: 10.1038/srep00856 23181188 PMC3504474

[46] Villamuelas M, Serrano E, Espunyes J, Fernández N, López-Olvera JR, Garel M, et al. Predicting herbivore faecal nitrogen using a multispecies near-infrared reflectance spectroscopy calibration. PLoS One. 2017;12(4):1–15. doi: 10.1371/journal.pone.0176635 28453544 PMC5409079

[47] Jarque-bascuñana L, Bartolomé J, Serrano E, Espunyes J, Garel M, Alarcón JAC, et al. Near infrared reflectance spectroscopy analysis to predict diet composition of a mountain ungulate species. Animals. 2021;11(5). doi: 10.3390/ani11051449 34070176 PMC8158497

[48] Gálvez-Cerón A, Serrano E, Bartolomé J, Mentaberre G, Fernández-Aguilar X, Fernández-Sirera L, et al. Predicting seasonal and spatial variations in diet quality of Pyrenean chamois (Rupicapra pyrenaica pyrenaica) using near infrared reflectance spectroscopy. Eur J Wildl Res. 2013;59(1):115–21.

[49] Tolleson DR, Randel RD, Stuth JW, Neuendorff DA. Determination of sex and species in red and fallow deer by near infrared reflectance spectroscopy of the faeces. Small Ruminant Res. 2005;57(2–3):141–50.

[50] Santos JPV, Vicente J, Villamuelas M, Albanell E, Serrano E, Carvalho J, et al. Near infrared reflectance spectroscopy (NIRS) for predicting glucocorticoid metabolites in lyophilised and oven-dried faeces of red deer. Ecol Indic. 2014;45:522–8. Available from: 10.1016/j.ecolind.2014.05.021

[51] Keating MS, Stuth JW, Tolleson DR. Prediction of diet quality parameters of Rocky Mountain Elk via near infrared reflectance spectroscopy (NIRS) fecal profiling. Proc Tex Chapt, Wild Soc College Station. 2005;16. Available from: http://scholar.google.com/scholar?hl=en&btnG=Search&q=intitle:PREDICTION+OF+DIET+QUALITY+PARAMETERS+OF+ROCKY+MOUNTAIN+ELK#0

[52] Brooks J, Anderson M, Urness PT. Infrared Reflectance Analysis of Forage Quality for Elk. 2010;48(1):254–8.

[53] Tolleson D, Halstead L, Howery L, Schafer D, Prince S, Banik K. The Effects of a Rotational Cattle Grazing System on Elk Diets in Arizona Piñon-Juniper Rangeland. 2012.34(1),19–25.

[54] Arnon A, Landau SY, Izhaki I, Malkinson D, Levy-Paz Y, Deutch-Traubman T, et al. A nirs-aided methodology to elucidate the nutrition of the endangered mountain gazelle (Gazella gazella) using samples of rumen contents from roadkills. Remote Sens (Basel). 2021;13(21):1–18.36817948

[55] Walker JW, Campbell ES, Lupton CJ, Taylor CA, Waldron DF, Landau SY. Effects of breed, sex, and age on the variation and ability of fecal near-infrared reflectance spectra to predict the composition of goat diets. J Anim Sci. 2007;85(2):518–26. doi: 10.2527/jas.2006-202 17235035

[56] Tolleson DR, Teel PD, Stuth JW, Strey OF, Welsh TH, Carstens GE. Fecal NIRS: Detection of tick infestations in cattle and horses. Vet Parasitol. 2007;144(1–2):146–52. doi: 10.1016/j.vetpar.2006.09.018 17097809

[57] Tigabu M, Felton AM. Multivariate calibration of near infrared spectra for predicting nutrient concentrations of solid moose rumen contents. Silva Fennica. 2018;52(1). doi: 10.14214/sf.7822

[58] Landau SY, Isler I, Dvash L, Shalmon B, Arnon A, Saltz D. Estimating the suitability for the reintroduced arabian oryx (Oryx leucoryx, Pallas 1777) of two desert environments by NIRS-aided fecal chemistry. Vol. 13, Remote Sensing. 2021.13(10) 1876; doi: 10.3390/rs13101876

[59] Greyling MD. (2010). Sex and age related distinctions in the feeding ecology of the African elephant. 2010. Thesis. http://hdl.handle.net/10539/7489

[60] Chaitae A, Rittiron R, Gordon IJ, Marsh H, Addison J, Pochanagone S, et al. Shining NIR light on ivory: A practical enforcement tool for elephant ivory identification. Conserv Sci Pract. 2021;3(9):1–11.

[61] Espinoza EO, Baker BW, Moores TD, Voin D. Forensic identification of elephant and giraffe hair artifacts using HATR FTIR spectroscopy and discriminant analysis. Endanger Species Res. 2009;9(3):239–46.

[62] Prakash C, Sharma S, Singh R. Forensic Science International: Animals and Environments Species discrimination from blood traces using ATR FT-IR spectroscopy and chemometrics: Application in wildlife forensics. Forensic Science International: Animals and Environments [Internet]. 2023;3(December 2022):100060. Available from: 10.1016/j.fsiae.2022.100060

[63] Čepelka L, Jánová E, Suchomel J, Heroldová M. Use of nirs in wild rodents’ research: A review of timid beginnings. Remote Sens (Basel). 2021;13(16):1–13.36817948

[64] Tuomi MW, Muguzur FJA, Hoset KS, Soininen EM, Vesterinen E, Utsi TAa, et al. Novel frontier in wildlife monitoring: identification of small rodent species from faecal pellets using Near-Infrared Reflectance Spectroscopy (NIRS). Ecol and Evol. 2023; 13(3) e9857. doi: 10.1002/ece3.9857 PMC10024998 36950367

[65] Johnson-Ulrich L, Vance CK, Kouga AJ, Willard ST. Fecal Near Infrared Reflectance FNIR Spectroscopy for discrimination of species and gender for Amur leopards and snow leopards. NIR 2013 - 16th International Conference on Near Infrared Spectroscopy [Internet]. 2013;:495–505. Available from: http://citeseerx.ist.psu.edu/viewdoc/download?doi=10.1.1.654.6433&rep=rep1&type=pdf#page=19

[66] Rothman JM, Chapman CA, Hansen JL, Cherney DJR, Pell AN. Rapid assessment of the nutritional value of foods eaten by mountain gorillas: Applying near-infrared reflectance spectroscopy to primatology. Int J Primatol. 2009;30(5):729–42.

[67] Rojas H, Rodriguez-Fernandez J. Near infrared spectroscopy in hairs: a rapid and non-invasive identification of species and sex in primates. 17th International Conference on Near Infrared Spectroscopy. 2015.

[68] Kinoshita K, Kuze N, Kobayashi T, Miyakawa E, Narita H, Inoue-Murayama M, et al. Detection of urinary estrogen conjugates and creatinine using near infrared spectroscopy in Bornean orangutans (Pongo Pygmaeus). Primates. 2016;57(1):51–9. doi: 10.1007/s10329-015-0501-3 26561334

[69] André J, Lawler IR. Near infrared spectroscopy as a rapid and inexpensive means of dietary analysis for a marine herbivore, dugong Dugong dugon. Mar Ecol Prog Ser. 2003;257:259–66.

[70] Lawler IR, Aragones L, Berding N, Marsh H, Foley W. Near-infrared reflectance spectroscopy is a rapid, cost-effective predictor of seagrass nutrients. J Chem Ecol. 2006;32(6):1353–65. doi: 10.1007/s10886-006-9088-x 16770723

[71] Kaneko H, Lawler IR. Can near infrared spectroscopy be used to improve assessment of marine mammal diets via fecal analysis? Marine Mammal Sci. 2006;22(2):261–275.

[72] Moore BD, Lawler IR, Wallis IR, Beale CM, Foley WJ. Palatability mapping: A koala’s eye view of spatial variation in habitat quality. Ecology. 2010;91(11):3165–76. doi: 10.1890/09-1714.1 21141178

[73] Foley WJ. Near infrared reflectance spectroscopy in ecological studies of plant–animal interactions. Spectroscopy Eur. 2009;21(5):6–9.

[74] Billing J. Kangaroo faeces: a reflection of kangaroo nutrition. Pest or Guest. 2007;182–4.

[75] Woolnough AP, Foley WJ. Rapid evaluation of pasture quality for a critically endangered mammal, the northern hairy-nosed wombat (Lasiorhinus krefftii). Wildlife Res. 2002;29(1):91–100.

[76] Power AC, Chapman J, Chandra S, Roberts JJ, Cozzolino D. Illuminating the flesh of bone identification–An application of near infrared spectroscopy. Vib Spectrosc. 2018;98:64–8.

[77] Santos-Rivera M, Feeney RZ, Julien AR, Guy E, Gillis A, Zhang HX, Kouba AJ, Vance CK. Gender discrimination using Near Infrared Reflectance (NIR) Spectroscopy in three caudate species. In: Proceedings of the 19th International Council for Near Infrared Spectroscopy Conference (NIR-2019), Gold Coast, Australia, 15–20 September 2019. 2020. p. 423–7.

[78] Zouboulis CC. The skin as an endocrine organ. Dermatoendocrinol. 2009;1(5):250–2. doi: 10.4161/derm.1.5.9499 20808511 PMC2836429

[79] Osisanwo FY, JAkinsola E.T, Awodele O, Hinmikaiye JO, Olakanmi O, Akinjobi J. Supervised Machine Learning Algorithms: Classification and Comparison. International J of Comp Trends and Tech. 2017;48(3):128–38.

[80] Benson IM, Helser TE, Marchetti G, Barnett BK. The future of fish age estimation: deep machine learning coupled with Fourier transform near-infrared spectroscopy of otoliths. Can of Fish Aqua Sci. 2023;80(9):1482–94.

[81] Hanzelik PP, Gergely S, Gáspár C, Győry L. Machine learning methods to predict solubilities of rock samples. J Chemom. 2020;34(2):1–13.

[82] Wolpert DH. The Lack of a Priori Distinctions between Learning Algorithms. Neural Comput. 1996;8(7):1341–90.

[83] Seaborn T, Goldberg CS, Crespi EJ. Drivers of distributions and niches of North American cold-adapted amphibians: evaluating both climate and land use. Ecological App. 2021;31(2). doi: 10.1002/eap.2236 33052615

[84] Sabatino SJ, Routman EJ. Phylogeography and conservation genetics of the hellbender salamander (Cryptobranchus alleganiensis). Con Genetics. 2009;10(5):1235–46.

[85] Building Predictive Models in R Using the Caret Package, J Stat Soft. 2008;28(5). doi: 10.18637/jss.v028.i05

[86] Kiemnec-Tyburczy KM, Watts RA, Gregg RG, von Borstel D, Arnold SJ. Evolutionary shifts in courtship pheromone composition revealed by EST analysis of plethodontid salamander mental glands. Gene. 2009;432(1–2):75–81. doi: 10.1016/j.gene.2008.11.007 19084057

[87] Antoniazzi MM, Mailho-Fontana PL, Nomura F, Azevedo HB, Pimenta DC, Sciani JM, et al. Reproductive behaviour, cutaneous morphology, and skin secretion analysis in the anuran Dermatonotus muelleri. iScience. 2022;25(4). doi: 10.1016/j.isci.2022.104073 35372815 PMC8968045

[88] Uchiyama M, Konno N. Hormonal regulation of ion and water transport in anuran amphibians. Vol. 147, General and Comparative Endocrinology. Academic Press Inc.; 2006. p. 54–61. 16472810 10.1016/j.ygcen.2005.12.018

[89] Largen W, Woodley SK. Cutaneous tail glands, noxious skin secretions, and scent marking in a terrestrial salamander (Plethodon shermani). Herpetologica. 2008;64(3):270–80.

[90] Hardman RH, Reinert LK, Irwin KJ, Oziminski K, Rollins-Smith L, Miller DL. Disease state associated with chronic toe lesions in hellbenders may alter anti-chytrid skin defenses. Sci Rep. 2023;13(1). doi: 10.1038/s41598-023-28334-4 36737574 PMC9898527

[91] Geng X, Wei H, Shang H, Zhou M, Chen B, Zhang F, et al. Proteomic analysis of the skin of Chinese giant salamander (Andrias davidianus). J Proteomics. 2015;119:196–208. doi: 10.1016/j.jprot.2015.02.008 25725404

[92] Clarke B. The natural history of amphibian skin secretions, their normal functioning and potential medical applications. Biol Rev Camb Philos Soc. 1997;72(3):365–79. doi: 10.1017/s0006323197005045 9336100

[93] Cei JM, Erspamer V. Biochemical Taxonomy of South American Amphibians by Means of Skin Amines and Polypeptides. Vol. 22. 1966. https://www.jstor.org/stable/1440763

[94] Woodhams DC, Ardipradja K, Alford RA, Marantelli G, Reinert LK, Rollins-Smith LA. Resistance to chytridiomycosis varies among amphibian species and is correlated with skin peptide defenses. Anim Conserv. 2007;10(4):409–17.

[95] Wang B, Zheng L, Liu DL, Ji F, Clark A, Yu Q. Using multi-model ensembles of CMIP5 global climate models to reproduce observed monthly rainfall and temperature with machine learning methods in Australia. Int of Clim. 2018;38(13):4891–902.

[96] Kiangala SK, Wang Z. An effective adaptive customization framework for small manufacturing plants using extreme gradient boosting-XGBoost and random forest ensemble learning algorithms in an Industry 4.0 environment. Machine Learning with App. 2021;4:100024.

[97] Gurumurthy S, Yu L, Zhang C, Jin Y, Li W, Zhang X, et al. Wildlife Poaching Prediction with Data and Human Knowledge. 2018. Available from: https://arxiv.org/abs/1805.05356

[98] Yun YH, Li HD, Deng BC, Cao DS. An overview of variable selection methods in multivariate analysis of near-infrared spectra. TrAC—Trends in Analytical Chemistry. 2019;113:102–15. Available from: 10.1016/j.trac.2019.01.018

[99] Chandrashekar G, Sahin F. A survey on feature selection methods. Com and Elect Eng. 2014;40(1):16–28.

[100] Au J, Youngentob KN, Foley WJ, Moore BD, Fearn T. Sample selection, calibration and validation of models developed from a large dataset of near infrared spectra of tree leaves. J Near Infrared Spectrosc. 2020;28(4):186–203.

[101] Wainer J, Cawley G. Nested cross-validation when selecting classifiers is overzealous for most practical applications. Expert Syst Appl. 2021;182. doi: 10.1016/j.eswa.2021.115222

[102] Vabalas A, Gowen E, Poliakoff E, Casson AJ. Machine learning algorithm validation with a limited sample size. PLoS One. 2019;14(11). doi: 10.1371/journal.pone.0224365 31697686 PMC6837442

[103] Cozzolino D. The sample, the spectra and the maths-The critical pillars in the development of robust and sound applications of vibrational spectroscopy. Molecules. 2020;25(16). doi: 10.3390/molecules25163674 32806655 PMC7466136

[104] R Core Team. R: A language and environment for statistical computing. [Internet]. Vienna, Austria: R Foundation for Statistical Computing; 2022. Available from: https://www.r-project.org/

[105] RStudio Team. RStudio: Integrated Development for R [Internet]. Boston, MA: R Studio; 2022. Available from: https://www.rstudio.com/

[106] Bischl B, Lang M, Kotthoff L, Schiffner J, Richter J, Studerus E, et al. mlr: Machine Learning in R [Internet]. Vol. 17, J of Machine Learning Res. 2016. Available from: https://github.com/mlr-org/mlr

[107] Chen T, He T. xgboost: eXtreme Gradient Boosting. 2017.

[108] Jerome A, Hastie T, Tibshirani R, Tay K, Simon N, Yang J. Package ‘ glmnet ‘ R topics documented: 2022.

[109] Stevens A, Ramirez-Lopez L. An introduction to the prospectr package. 2014. https://antoinestevens.github.io/prospectr/

[110] Kursa M. Package “Boruta”: Wrapper Algorithm for All Relevant Feature Selection [Internet]. 2018. Available from: https://notabug.org/mbq/Boruta/

[111] Dematheis F, Walter MC, Lang D, Antwerpen M, Scholz HC, Pfalzgraf MT, et al. Machine Learning Algorithms for Classification of MALDI-TOF MS Spectra from Phylogenetically Closely Related Species Brucella melitensis, Brucella abortus and Brucella suis. Microorganisms. 2022;10(8). doi: 10.3390/microorganisms10081658 36014076 PMC9416640

[112] SAS Institute Inc. JMP. Cary, NC: SAS Institute Inc.; 2023.

[113] Zhang X, Yang J, Lin T, Ying Y. Food and agro-product quality evaluation based on spectroscopy and deep learning: A review. Vol. 112, Trends in Food Science and Technology. Elsevier Ltd; 2021. p. 431–41.

[114] Brand W, Wells AT, Smith SL, Denholm SJ, Wall E, Coffey MP. Predicting pregnancy status from mid-infrared spectroscopy in dairy cow milk using deep learning. J Dairy Sci. 2021;104(4):4980–90. doi: 10.3168/jds.2020-18367 33485687

[115] Le BT. Application of deep learning and near infrared spectroscopy in cereal analysis. Vib Spectrosc. 2020;106. doi: 10.1016/j.vibspec.2019.103009

