# Supplementary material for: Enhancing predictive performance for spectroscopic studies in wildlife science through a multi-model approach: A case study for species classification of live amphibians
Source: PLoS Comput Biol. 2024 Feb 14;20(2):e1011876. doi: 10.1371/journal.pcbi.1011876 (PMC10898777; doi:10.1371/journal.pcbi.1011876)
Supplement: S4 Document — (DOCX) [file pcbi.1011876.s004.docx]

The numbers in brackets [lower limit, upper limit] following tuning parameters specify the parameter search space used to tune each model.

1. **Generalized Linear Model with elastic net regression (GLMnet):** a supervised linear regression method traditionally used for making causal inferences but has more recently been utilized as a supervised machine learning algorithm for predictive applications. GLM makes predictions based on the maximum likelihood estimates of predictor variables that best explain the response variable [1]. GLMnet is a penalized regression method that uses elastic net regularization, a method which utilizes a combination of lasso and ridge regression. Ridge regression will not drive the coefficients for variables down to zero, whereas lasso can, which allows it to function as a feature selection technique (i.e., the coefficients of non-important variables are driven to zero; therefore, variables that do not contribute to the regression are removed from the model [2]).
   1. “s” [0.0, 0.1] by 0.02: specifies the lower and upper penalty parameter, lambda.
   2. Alpha [0, 1] by 0.2: specifies the lower and upper limits for the elastic net mixing parameter. This value determines how much penalty is applied using the ridge (alpha = 0) or lasso (alpha = 1) penalty.
2. **K-Nearest Neighbors (KNN)**: a supervised machine learning algorithm that predicts the class of an observation based on the majority-class of K- nearest neighbors, K being the number of neighbors designated in the model statement that are included in the poll [3].
   1. “K” [1,5]: specifies the lower and upper limits of “K” nearest neighbors when making predictions.
3. **Principal Component Analysis-Linear Discriminant Analysis (PCA-LDA)**: a combined, unsupervised-supervised machine learning algorithm that has been used extensively as a dimensionality-reducing technique for discriminating observations amongst discrete classes. PCA-LDA functions by transforming features to an altered dimensional plane that separates and maximizes the distance between classes using Euclidian or Mahalanobis distance by maximizing the ratio of within-class/between-class variance [4]. Note: one major constraint to LDA is that it cannot contain more features than observations and it does not run when collinearity between features is high; PCA is a dimensionality reduction technique mitigating both of those concerns.
   1. Principal components [1,100]: specifies the lower and upper limits for the number of principal components to be utilized when making predictions.
4. **Partial Least Squares (PLS)**: a supervised machine learning algorithm that functions as both a reduction and discrimination technique, similarly to LDA. PLS generates orthogonal, latent components from the features present in the dataset which maximizes the covariance among features. PLS components represent new predictor variables that contain maximal information that may then be applied to either classify or regress the response variable [5].
   1. Ncomp [1,100]: specifies the lower and upper limits for the number of components or factors to be utilized for making predictions.
5. **Random Forest (RF)**: a supervised machine learning algorithm that applies an ensemble approach when placing predictions based on repetitive splitting of features (represented as branches) at random to split nodes into decision trees. Each independent tree casts a vote and the class receiving the most votes by the forest of trees are chosen in the resampling [6].
   1. Mtry [2,10]: specifies the lower and upper number of variables randomly sampled as candidates at each split.
6. **Support Vector Machine (SVM)**: a linear, supervised machine learning algorithm which plots all observations on a hyperplane, which is a multi-dimensional plane comprised of all predictor features. Classification decisions are based on tuned, optimal decision boundaries (i.e., support vectors) used to divide the various variable classes. The support vectors are informative datapoints that influence the hyperplane to maximize the margin between classes in order to ultimately build the SVM and predict classes of new observations. Parameter tuning helps to balance the margin maximization and loss to improve SVM model performance [7].
   1. Cost [0,6]: specifies the lower and upper limits for the penalty applied for misclassifying an observation.
   2. Gamma [-5,1]: specifies the upper and lower limit for how the decision boundary is reshaped through the process of categorizing and clustering similar samples, based on the inverse of the radius of influence for the selected support vectors.
7. **Extreme Gradient Boosting (XGBoost)**: a supervised machine learning algorithm that employs an ensemble of trees to make a prediction, and in difference to traditional methods that optimize parameters using Euclidian distances, boosted models train the model additively as more trees are added to fit and correct prediction errors observed in previous iterations. The term gradient boosting comes from the concept that improved models are generated by attempts to minimize loss (i.e., error) in subsequent trees. The ensemble of trees is used to make a final prediction [8].
   1. Nrounds [100,600]: specifies the lower and upper limit for the number of trees that are built iteratively for making predictions.
   2. Max_depth [1,100]: specifies how deep the trees can grow or how many levels of nodes may be present.
   3. Eta: [0.1, 0.75]: specifies the learning rate; with each subsequent round evaluates at gradients and follows gradients based on distances between classes.
   4. Gamma [0,5]: specifies the regularization (lasso or ridge regression) used to prevent overfitting.
   5. Colsample_bytree [0.1,1]: specifies the lower and upper limit for the number of features (variables) used to train each tree (similar to “mtry” used in random forest models).
   6. Colsample_bylevel [0.1,1]: specifies the lower and upper limit for the fraction of trees randomly selected to train each node in each tree (i.e., the percentage of features selected at random to make the splits on any given level).

**REFERENCES**

1. Arnold KF, Davies V, de Kamps M, Tennant PWG, Mbotwa J, Gilthorpe MS. Reflection on modern methods: Generalized linear models for prognosis and intervention-theory, practice and implications for machine learning. Int J Epidemiol. 2020 Dec 1;49(6):2074–82.

2. Jerome A, Hastie T, Tibshirani R, Tay K, Simon N, Yang J. Package ‘ glmnet ’ R topics documented : 2022;

3. Batista G, Furtado Silva D. How k-Nearest Neighbor Parameters Affect its Performance [Internet]. 2009. Available from: http://www.icmc.usp.br/

4. Tharwat A, Gaber T, Ibrahim A, Hassanien AE. Linear discriminant analysis: A detailed tutorial. AI Communications. 2017;30(2):169–90.

5. Rosipal R, Krämer N. Overview and Recent Advances in Partial Least Squares. 2006.

6. Breiman L. Random Forests. Vol. 45. 2001.

7. Wang L, Springer B. Support Vector Machines: Theory and Applications. 2005.

8. Chen T, Guestrin C. XGBoost: A scalable tree boosting system. In: Proceedings of the ACM SIGKDD International Conference on Knowledge Discovery and Data Mining. Association for Computing Machinery; 2016. p. 785–94.
